# Recreational Cannabis Use and Risk of Prescription Opioid Overdose: Insights from Pediatric Inpatients

**DOI:** 10.7759/cureus.11058

**Published:** 2020-10-20

**Authors:** Amaya Pankaj, Kosisochukwu Oraka, Emmanuelle J Caraballo-Rivera, Munazza Ahmad, Shaheer Zahid, Sadaf Munir, Gayathri Gurumurthy, Onose Okoeguale, Shikha Verma, Rikinkumar S Patel

**Affiliations:** 1 Pediatric Medicine, Jawaharlal Institute of Postgraduate Medical Education and Research, Puducherry, IND; 2 Medicine, Vinnytsia National Medical University, Vinnytsia, UKR; 3 Medical Sciences, Ponce Health Sciences University, Ponce, PRI; 4 Medicine, Lahore Medical & Dental College, Lahore, PAK; 5 Psychiatry, Saint James School of Medicine, Park Ridge, USA; 6 Psychiatry, Jersey Shore University Medical Center, Neptune, USA; 7 Internal Medicine, Shadan Institute of Medical Sciences, Hyderabad, IND; 8 Psychiatry, Vinnytsia National Medical University, Vinnytsia, UKR; 9 Psychiatry and Behavioral Sciences, Rogers Behavioral Health, Kenosha, USA; 10 Psychiatry and Behavioral Sciences, Oklahoma State University, Tulsa, USA; 11 Psychiatry, Griffin Memorial Hospital, Norman, USA

**Keywords:** prescription opioid, opioid overdose, adolescents, substance use, cannabis use, marijuana use, opioid use disorders

## Abstract

Objectives

Our first goal is to evaluate the prevalence of hospital admissions for prescription opioid overdose (POD) in pediatric inpatients, and next goal is to measure the independent association between cannabis use disorders (CUD) and POD.

Methods

We used the nationwide inpatient sample (NIS) and included 27,444,239 pediatric inpatients (age ≤ 18 years), and 10,562 (0.04%) were managed primarily for POD. The odds ratio (OR) of the association of variables in POD inpatients was measured using the binomial logistic regression model that was adjusted for demographic confounders and psychiatric comorbidities.

Results

Adolescents have higher odds (OR 10.75, 95% CI 10.16-11.36) of POD-related hospitalization compared to children ≤ 12 years. Whites formed the significant proportion (67%), and those from low-income families (<50th percentile) had higher likelihood for POD-related hospitalization. The most prevalent psychiatric comorbidities were mood disorders (44.3%) and anxiety disorders (14.6%). Prevalent comorbid substance use disorders (SUDs) included cannabis (14.2%), tobacco (13.1%), and opioid (9.4%). A higher odds of association with POD-related hospitalizations were seen in pediatric inpatients with comorbid opioid (OR 8.79, 95% CI 8.08-9.56), tobacco (OR 1.58, 95% CI 1.47-1.70), and cannabis (OR 1.68, 95% CI 1.57-1.81) use disorders.

Conclusion

The prescription opioid is a bridge to opioid abuse/dependence, thereby increasing the risk of other SUDs like tobacco (by 58%) and cannabis (by 68%). Regulating the easy availability of prescription opioids and also improving the existing prescription trends are an essential way to reduce this problem. Finally, awareness and counseling are recommended strategies for harm reduction/rehabilitation among the pediatric population.

## Introduction

A widespread emphasis and discussion over adequate assessment and treatment of pain in children are, over the past decade, resulting in a surge in the prescription of opioids in pediatric emergency departments (ED). Concurrently, opioid overdose-related hospitalizations and the non-medical use of opioids among adolescents have been a growing health concern [1]. The tendency of adolescents to engage in high-risk behavior and experimentation must be taken into account as substance and alcohol abuse, and violence tend to co-occur [2]. According to the national survey on drug use and health, prescription analgesics were the second most commonly abused substances by adolescents and young adults [1].

The opioid prescription seems to be influenced by factors like race, gender, and insurance. This trend mimics the adult population in terms that black patients seem to receive fewer opioid prescriptions, a trend which may be attributed to lack of access to medical care and medications due to cost and lesser availability of pharmacy [3,4]. Thereby, white children seem to form a sizable proportion of the opioid-related hospitalizations. Also, there is an increased tendency to prescribe opioids in patients on private insurance, which may be due to improving patient satisfaction, but again this exposes the child to easier availability of opioids for misuse [5]. Though pain assessment and relief are of utmost importance in the pediatric population, studies have shown that inadequate pain relief in this age group can have a worse prognosis, but also making easier availability of opioids can lead to accidental prescription opioid overdoses, poisoning, and misuse, which should be given greater attention [6,7].

We used the largest inpatient database from the United States (US) hospitals to evaluate the prevalence of hospital admissions for prescription opioid overdose (POD) in pediatric inpatients and to measure the independent association between cannabis use disorders (CUD) and POD-related hospitalizations. Also, we studied the differences in demographic patterns and comorbid psychiatric and substance use disorders (SUDs) for POD with CUD versus without CUD.

## Materials and methods

Data source

We used the nationwide inpatient sample (NIS) from the healthcare cost and utilization project (HCUP), which is the largest hospital-based dataset in the United States. The NIS constitutes patient discharge diagnosis information (clinical and non-clinical) from 4,400 hospitals across the 44 states in the Unted States. Diagnostic information in the NIS is identified using the international classification of diseases, ninth edition (ICD-9) codes [8].

Inclusion criteria

We conducted a retrospective analysis of the NIS (2010 to 2014) to identify all non-elective/emergency admissions for pediatric patients (age 18 and below) with a primary ICD-9 discharge diagnosis for POD (965.00, 95.01, and 965.09). These patients were further grouped by comorbid CUD identified by ICD-9 codes (304.30, 304.31, 304.32, 305.20, 305.21, and 305.22). Patients coded as “in remission” from cannabis abuse or dependence were excluded from our analysis to maintain the uniformity of the study cohort.

Variables

Demographic variables including age, sex, race, and median household income were evaluated in this study. Psychiatric comorbidities (anxiety disorder, mood disorders, psychotic disorders, and suicidality) and SUDs (opioid, amphetamine, and cocaine) were identified using the ICD-9 diagnosis codes [9].

Statistical analysis

The analyses were conducted using the statistical package for the social sciences (SPSS) version 26 (IBM Corp., Armonk, NY). We utilized bivariate descriptive analysis to compare demographics and psychiatric comorbidities and SUDs in patients with POD and without POD (non-POD) cohorts followed by binomial logistic regression analyses to evaluate the demographic predictors and risk factors for POD-related hospitalization. Next, we evaluated the age-wise odds for POD-related hospitalization in patients with CUD compared to patients without CUD (non-CUD). The regression models were adjusted for demographics confounders (age, sex, race, and median household income) and psychiatric comorbidities. Lastly, we compared CUD and non-CUD cohorts using bivariate descriptive analysis followed by binomial logistic regression analyses to identify the demographic predictors and risk factors in POD patients with CUD. Due to the large sample size and multiple comparisons of data, statistical significance was set at p value < 0.01.

Ethical approval

Unique subject identifiers were used to secure patient information. The use of the publicly available de-identified NIS data of the HCUP, according to the department of health and human services does not require approval from an institutional review board [8].

## Results

We analyzed a total of 27,444,239 pediatric hospitalizations, and 10,562 (0.04%) were primarily POD-related hospitalization. Adolescents have 11 times higher odds (95% CI 10.16-11.36) of POD-related hospitalization compared to children under 11 years, and whites form about two-third proportion (67%) of the POD-cohort. POD-related hospitalizations were seen in children from low- to middle-income families by 1.3 times compared to those from high-income families (above 75th percentile).

The most prevalent psychiatric comorbidities were mood disorders and anxiety disorders, and children diagnosed with mood disorders had 4.6 times higher odds (95% CI 4.40-4.92) of POD-related hospitalization. The most prevalent comorbid SUDs include cannabis (14.2%), tobacco (13.1%), and opioid (9.4%) use disorders. There exists a higher odds of association with POD-related hospitalizations in children with comorbid opioid (OR 8.79, 95% CI 8.08-9.56), tobacco (OR 1.58, 95% CI 1.47-1.70), and cannabis (OR 1.68, 95% CI 1.57-1.81) use disorders as shown in Table 1.

**Table 1:** Suspected risk factors for prescription opioid overdose-related hospitalization. The proportion of non-POD and POD patients was obtained using cross-tabulation, and the odds ratio was generated by the binomial logistic regression model and was adjusted for all variables mentioned. POD: Prescription opioid overdose.

Variable	Non-POD (%)	POD (%)	Odds ratio	95% Confidence interval	p value
Total inpatients	27,433,677	10,562	-	-	-
Age at admission					
<11 years	88.9	26.6	Reference		
12–18 years	11.1	73.4	10.75	10.16–11.36	<0.001
Sex					
Male	51.0	48.0	Reference		
Female	49.0	52.0	0.87	0.83–0.91	<0.001
Race					
White	51.3	67.0	Reference		
Black	15.8	12.4	0.61	0.57–0.65	<0.001
Hispanic	21.4	12.5	0.59	0.55–0.63	<0.001
Other	11.5	8.0	0.84	0.77–0.91	<0.001
Median household income					
0–25^th ^percentile	29.0	28.8	1.29	1.21–1.37	<0.001
26^th^–50^th ^percentile	25.1	27.1	1.35	1.27–1.44	<0.001
51^st^–75^th^ percentile	24.6	25.2	1.29	1.21–1.37	<0.001
76^th^–100^th ^percentile	21.4	18.9	Reference		
Comorbid psychiatric disorders					
Anxiety disorders	1.4	14.6	1.03	0.97–1.10	0.339
Mood disorders	3.0	44.3	4.65	4.40–4.92	<0.001
Suicidality	1.0	7.1	0.41	0.38–0.45	<0.001
Psychotic disorders	0.3	1.6	0.73	0.62–0.86	<0.001
Comorbid substance use disorders					
Tobacco	0.6	13.1	1.58	1.47–1.70	<0.001
Cannabis	0.6	14.2	1.68	1.57–1.81	<0.001
Opioid	0.1	9.4	8.79	8.08–9.56	<0.001
Amphetamine	0	1.0	0.66	0.53–0.82	<0.001
Cocaine	0	1.5	0.83	0.69–0.99	0.043

We also analyzed the suspected risk factors for CUD inpatients with POD. Out of the total 10,562 POD-related hospitalizations, 1,505 (14.2%) had comorbid CUD. About 92% of the cohort was constituted by adolescents who had two times higher odds (95% CI 1.56-2.44) of comorbid CUD compared to children under 11 years. Race-wise, whites formed the majority of the CUD cohort (75.2%), but the hispanics had higher odds (OR 1.14, 95% CI 0.92-1.40) of comorbid CUD compared to the other race/ethnicities. Among comorbid SUDs, there was a statistically significant rise in the use of opioid, tobacco, cocaine, and amphetamine as shown in Table 2.

**Table 2:** Suspected risk factors for cannabis use disorders in children with prescription opioid overdose. The proportion of non-CUD and CUD patients was obtained using cross-tabulation, and the odds ratio was generated by the binomial logistic regression model and was adjusted for all variables mentioned. CUD: Cannabis use disorders.

Variable	No CUD (%)	CUD (%)	Odds ratio	95% Confidence interval	p value
Total inpatients	9,058	1,505	-	-	-
Age at admission					
<11 years	29.7	7.8	Reference		
12–18 years	70.3	92.2	1.96	1.56–2.44	<0.001
Sex					
Male	46.0	59.9	Reference		
Female	54.0	40.1	0.55	0.48–0.63	<0.001
Race					
White	65.6	75.2	Reference		
Black	13.5	6.4	0.66	0.51–0.85	0.001
Hispanic	12.7	11.3	1.14	0.92–1.40	0.225
Other	8.2	7.1	0.82	0.62–1.07	0.135
Median household income					
0–25^th^ percentile	29.1	26.5	0.83	0.69–1.01	0.064
26^th^–50^th^ percentile	27.5	25.0	0.82	0.68–0.99	0.040
51^st^–75^th^ percentile	25.2	25.1	0.82	0.68–0.99	0.034
76^th^–100^th^ percentile	18.1	23.4	Reference		
Comorbid psychiatric disorders					
Anxiety disorders	13.7	20.3	1.13	0.95–1.33	0.174
Mood disorders	41.9	58.7	1.77	1.53–2.05	<0.001
Suicidality	6.8	8.9	1.21	0.97–1.52	0.097
Psychotic disorders	1.4	2.7	1.34	0.88–2.04	0.175
Comorbid substance use disorders					
Tobacco	9.6	33.7	3.01	2.58–3.51	<0.001
Opioid	6.7	25.4	2.78	2.35–3.30	<0.001
Amphetamine	0.4	4.6	5.79	3.64–9.23	<0.001
Cocaine	0.7	6.9	6.99	4.76–10.29	<0.001

## Discussion

In overall pediatric hospitalizations, the prevalence of POD was 0.04% and majorly in adolescents and whites. About, half of the POD-related hospitalizations had mood disorders, and the most prevalent substance used was cannabis (14.2%) and tobacco (13.1%). After controlling for potential confounders, CUD was associated with 68% increased odds for POD-related hospitalization.

Adolescents were at higher risk of POD-related hospitalizations compared to children under 11 years. This was consistent with findings of a study using the national poison data system (NPDS), which found that prescription opioid exposure was highest among teenagers [10]. This may be attributable to the thrill-seeking behavior, peer pressure, and accessibility to prescription opioids, which doubled the rates of POD among adolescents and young adults since 1994 [11]. Whites constituted about 67% of total POD-related hospitalizations in our study, which is possible as they may have greater access and probability of receiving prescription opioids during ED visits compared to minorities [12]. Another notable finding was that POD-related hospitalizations were higher in low- to middle-income families compared to those from high-income families, and a similar trend was seen in the adult population [7,13]. This may be attributable to the easier availability and cheaper cost of prescription opioids relative to other substances with abuse potential. Males seem to have a slightly higher risk than females, but the difference is not as stark as with other illicit substances, which may be due to the fact that females are more likely to be prescribed an opioid and also the perception that abusing prescription medication is “safer” than abuse of other substances [14].

Our study found that there is a higher risk of psychiatric comorbidities in pediatric inpatients with POD. The most common psychiatric conditions associated are mood disorders and anxiety disorders, which are consistent with the study using the data from the national epidemiologic study on alcohol and related conditions (NESARC). This explains the bidirectional association between POD and psychiatric comorbidities, where adolescents tend to use prescription opioids to alleviate their mood symptoms. We also found that POD-related hospitalizations had comorbid SUDs with opioids, cannabis, and tobacco, with cannabis being the most prevalent substance used due to the rising use of cannabis among the pediatric population [15]. Also, an inpatient study found a higher prevalence of tobacco (36.8%) and cannabis (28.5%) use disorders among pediatric patients hospitalized for heroin overdoses [16].

There was a unique trend in risk factors, epidemiological outcomes, psychiatric comorbidities, and SUDs in the pediatric inpatients with CUD among POD-related hospitalizations. CUD was majorly seen in adolescents and males, which may be due to the reduced perception of risk and easier access to cannabis [17]. Though data from the national surveys on drug use and health suggests blacks form the majority having CUD in adults, in our study white children formed about three-fourths of POD-related hospitalizations. Yet, when compared with whites, hispanics with POD had 14% higher odds of comorbid CUD [18]. Pediatric inpatients with POD from high-income families were more likely to have CUD. This is in accordance with a study by Trim et al., which found that adolescents living in an affluent neighborhood are more likely to abuse substances due to lesser supervision [19].

Among cannabis users, there exists a significantly high risk of comorbid SUDs with higher odds of using cocaine (by seven times), amphetamines (by six times), and tobacco and opioids (by three times each) [20,21]. Mood and anxiety disorders, suicidal behaviors, and psychotic disorders were significantly associated with pediatric POD inpatients having CUD [22]. Youth with CUD at an earlier age have a greater risk of long-lasting cognitive impairment and several psychiatric and medical conditions [23-28].

Regulating the easy availability of prescription opioids and also improving the existing prescription trends is an essential way to reduce the problem of the current opioid epidemic in the United States. In the current opioid crisis, there is improved vigilance on prescribed opioids as compared to before, and the overall number of prescribed opioids is trending down. It is important that providers continue to regulate prescribed opioids. Parents and guardians should also understand the importance of safe storage of prescriptions, thereby keeping the medications out of reach from children [29]. It is imminent to engage youth and families in psychoeducation, harm reduction strategies, services provided in the community to drop extra medications, and if there are concerns for opioid abuse/dependence, providing individuals and families with naloxone spray. Earlier onset of SUDs is associated with long-term implications such as declining functioning due to ongoing financial, social, and occupational challenges and continued mental health and SUDs. The United States has the largest rates of an opioid burden for both males (968.6 disability-adjusted life years [DALYs] per 100,000) and females (565.7 DALYs per 100,000), and years of life lost (YLL) is also high at 265 per 100,000 [30].

There were a few limitations to our study. First, these findings may be underreported, thereby making it unrepresentative of the real burden of POD-related hospitalizations across the United States due to the nature of an administrative database that was used in this study. Second, we examined pooled data and year-by-year trend data, due to which we cannot rule out the possibility that outpatients and re-hospitalizations across different healthcare settings may not be represented. We used the NIS that enabled us to get a national representative sample of POD inpatients, and the large sample size also increased the power of our study. We analyzed trends of comorbid CUD in POD inpatients, which gave us a unique picture and increased external validity. Also, the findings of this study are relevant to understand the burden of POD, which can help to adequately understand and strategically devise specific intervention plans to control this public health problem.

## Conclusions

POD-related hospitalizations were observed to have a higher prevalence among white adolescents from affluent families. There are increased risks for other coexisting SUDs, most commonly with CUD and mental health concerns such as anxiety and mood disorders. Decreasing prescription opioids, parental education, youth awareness, understanding, and counseling are the recommended strategies for harm reduction and rehabilitation among the at-risk pediatric populations. Continued opioid abuse/dependence is associated with continued psychiatric and substance use challenges in adulthood. Opioid abuse/dependence is related to increased disease burden causing high DALYs and YLLs in the United States. Regulating the easy availability of prescription opioids and also improving the existing prescription trends are an essential way to reduce this problem.
